# DGAT1 Drives Racially Divergent Fibroblast Activation via ERK1/2-Dependent Tumorigenic Signaling in Prostate Cancer

**DOI:** 10.1158/2767-9764.CRC-25-0701

**Published:** 2026-06-04

**Authors:** Sathyavathi ChallaSivaKanaka, Mamatha Kakarla, Renee E. Vickman, Yana Filipovich, Philip Fitchev, Md Maksudul Alam, Md Niaz Morshed, Pooja Talaty, Brian T. Helfand, David Price, Simon W. Hayward, Susan E. Crawford, Omar E. Franco

**Affiliations:** 1 Department of Surgery, NorthShore University HealthSystem, Evanston, Illinois.; 2 Pritzker School of Medicine, University of Chicago, Chicago, Illinois.; 3 Department of Biochemistry and Molecular Biology, Louisiana State University Health Shreveport, Shreveport, Louisiana.; 4 Feist-Weiller Cancer Center, Louisiana State University Health Shreveport, Shreveport, Louisiana.

## Abstract

Lethal prostate cancer disproportionately affects African American (AA) men, who experience a higher incidence and earlier onset compared with European American (EA) men, reflecting a persistent biological and clinical disparity. Recent studies have implicated race-associated differences in lipid metabolic reprogramming as key contributors to this disparity. We recently identified carcinoma-associated fibroblasts (CAF) as a major stromal component that mediates racial disparities in tumor progression. Here, we demonstrate that lipid-laden CAFs from AA patients (AA^CAF^) display enhanced protumorigenic properties compared with CAFs from EA patients (EA^CAF^). Lipid droplet (LD) biogenesis and storage analysis revealed a robust diacylglycerol O-acyltransferase 1 (DGAT1) enzyme-dependent LD accumulation in AA^CAF^. Ectopic DGAT1 expression in benign fibroblasts induced CAF markers’ (FAP1 and αSMA) expression linked to fibroblast activation, altered the secretome, and significantly enhanced prostate cancer cells’ growth *in vivo*. Integrative transcriptomic and secretome analyses identified novel DGAT1-regulated genes involved in CAF linked to metabolism, cell–cell communication, motility, and angiogenesis, mediated mainly through ERK1/2 signaling activation. Pharmacologic DGAT1 inhibition suppressed these pathways and elicited racially distinct regulation of tumor-promoting mediators, including BDNF, VEGF, and TSP1. Mechanistic experiments show that functionally, lipid-laden CAFs from AA patients exhibit increased fibroblast activation and a secretory phenotype with greater protumor activity compared with CAFs from EA patients. Collectively, these findings reveal that DGAT1 is a pivotal enzymatic regulator of fibroblast activation and lipid-driven remodeling in the prostate cancer tumor microenvironment of AA men. Targeting DGAT1 represents a promising strategy to disrupt metabolic–stromal cross-talk and mitigate race-associated disparities in prostate cancer progression.

**Significance:**

This study highlights DGAT1-driven lipid accumulation in CAFs from AA patients with prostate cancer as a key driver of fibroblast activation and tumor-promoting roles contributing to racial disparities. Targeting DGAT1 disrupts lipid-mediated cancer–stroma interactions, offering a new therapeutic strategy to reduce aggressive prostate cancer in AA men.

## Introduction

African American (AA) men experience a disproportionately higher incidence and mortality from prostate cancer compared with European American (EA) men (1–4). Although socioeconomic factors, access to healthcare, and screening practices contribute to this disparity, epidemiologic and molecular studies increasingly demonstrate that these variables are insufficient to fully explain the magnitude of the difference, suggesting that underlying biological mechanisms also contribute significantly to prostate cancer risk and progression (5–8). Ancestry-associated factors influencing genetic and epigenetic alterations, tumor metabolism, and the tumor microenvironment (TME) are recognized as critical contributors to racial disparities, including in prostate cancer (7, 9–12). However, the molecular circuitry linking ancestry to TME remodeling and tumor progression remains poorly defined.

Rather than serving as passive structural elements, carcinoma-associated fibroblasts (CAF) orchestrate paracrine signaling networks that directly influence epithelial tumor cell behavior (13–15). We previously demonstrated that CAFs derived from AA patients (AA^CAF^) exhibit a secretory phenotype that enhances tumorigenicity, promotes neoangiogenesis, and increases inflammatory cell recruitment compared with CAFs derived from EA patients (EA^CAF^; ref. 9). Our findings also revealed brain-derived neurotrophic factor (BDNF), a member of the neurotrophin family with established roles in cell survival, differentiation, and guidance within the central and peripheral nervous systems, as a potential mediator of CAF–cancer cell cross-talk. Racial differences in BDNF levels have been reported in metabolic and neurodegenerative disorders, suggesting that ancestry-linked regulatory mechanisms may influence BDNF expression (16, 17). These findings implicate stromal reprogramming as a biologically relevant contributor to racial disparities in prostate cancer. Yet, the upstream metabolic drivers and downstream signaling mediators responsible for this ancestry-associated CAF phenotype remain unknown.

Lipid metabolic rewiring represents a central adaptive mechanism in both tumor and stromal cells. In adipocytes, excess fatty acids (FA) are esterified and stored in lipid droplets (LD) to prevent lipotoxic stress (18). In contrast, nonadipocytic stromal cells exposed to lipid-rich environments undergo a phenotypic transition from quiescence to activation, characterized by enhanced extracellular matrix remodeling and protumorigenic signaling. We previously demonstrated that patient-derived CAFs regulate lipid homeostasis through coordinated control of the lipogenic enzyme diacylglycerol O-acyltransferase 1 (DGAT1) and the lipolytic cofactor pigment epithelium-derived factor (PEDF), thereby facilitating sequestration of excess free FAs (FFA) into LDs (19). This metabolic buffering mechanism prevents lipotoxicity while simultaneously supporting a protumorigenic secretory phenotype. Importantly, AA men exhibit increased prostatic total FA and FFA levels (20), which are associated with more aggressive disease and poorer outcomes, suggesting that lipid availability may selectively amplify stromal metabolic adaptations in this population.

In the present study, we define the molecular mechanisms driven by elevated stromal DGAT1 in AA^CAF^ and delineate the downstream consequences for TME remodeling and cancer cell tumorigenicity. Our findings identify stromal DGAT1 as a key mediator of ancestry-associated differences in prostate cancer biology and suggest a mechanistic link between lipid metabolic reprogramming in CAFs and racial disparities in disease progression.

## Materials and Methods

### Cell culture and reagents

Upon written informed patient consent and approval from the institutional ethical committee [NorthShore University Health System (NSUHS) Institutional Review Board (IRB)-approved collection via the NorthShore Comprehensive Urologic Disease Biorepository and Database], deidentified human prostatic tissue samples were obtained from patients with prostate cancer undergoing robot-assisted laparoscopic prostatectomy (RALP). The studies were conducted in accordance with recognized ethical guidelines (e.g., Declaration of Helsinki, CIOMS, Belmont Report, and US Common Rule). Inclusion criteria for patients: males, between the ages of 50 and 90 years old, self-reported as AA (*n* = 9) or EA (*n* = 8). CAFs were isolated and validated for their *in vivo* tumor-promoting activity using previously optimized methods before being used for the proposed experiments (patient details are provided in Supplementary Table S1; ref. 9). The entire initial sample for the study was retained, and relevant data were collected for each participant. BPH1 and BHPrS1 cells from our stocks (21, 22) and human prostate cancer cell lines, LNCaP (RRID: CVCL_0395; CRL-1740) and PC-3 (CRL-1435; RRID: CVCL_0035), purchased from the ATCC were cultured according to ATCC recommendations. Cultures of low-passage cells (*P* < 25) were used for the experiments. All cells were authenticated by short tandem repeat profiling (ATCC) and routinely screened for *Mycoplasma* using the MycoAlert Kit (Lonza Inc.). Cell treatments include the following: 100 μmol/L oleic acid (OA; O3008, Sigma-Aldrich), 1 μmol/L DGAT1 inhibitor (A-922500, Cayman Chemical), or ERK1/2 inhibitor U0126 (Thermo Fisher Scientific 50-195-887).

### Plasmid constructs and cell line generation

A DGAT1-expressing plasmid (OHU 28798) was obtained from GenScript and subcloned into pENTR1A-GFP-N2 (FR1; RRID: Addgene_19364; a gift from Eric Campeau and Paul Kaufman, Addgene) using the In-Fusion Snap Assembly Bundle (638945, Takara). The entry clone, pENTR1A-*DGAT1*-GFP-N2, was cloned into pLenti CMV Blast DEST (706-1; RRID: Addgene_17451) to generate pLenti CMV Blast vectors using the Gateway LR Clonase reaction (11791019, Invitrogen). DGAT1 lentivirus was produced by stable transfection of 4.3 μg of Lentiviral expression vector and 1 μg/μL of ViraPower Lentiviral Packaging Mix (K497500, Invitrogen) into 293FT cells (CRL-1573), (RRID: CVCL_6911) purchased from the ATCC, in a 10-cm dish with Lipofectamine 3000 reagent (L3000-001, Invitrogen) according to the manufacturer’s instructions. Viral supernatants were collected 48 hours after transfection, passed through a 0.2-μm filter, and stored at −80°C. Cells were transduced in the presence of 4 μg/mL polybrene (Sigma-Aldrich) for 12 hours followed by 2 μg/mL blasticidin (ant-bl-05, InvivoGen) selection for 1 week.

### LD quantification

#### Flow cytometry

Cells were harvested with 0.25% trypsin-EDTA (Thermo Fisher Scientific) and washed with PBS (×3), resuspended in PBS, and kept on ice prior to FACS analysis. After setting both side scatter (SSC) and forward scatter, the voltages and compensation between scatters were set to determine the scale of control samples. Once optimized, three different gates were generated to assess lipid content and granularity.

### Staining

Nile Red and Oil-Red-O (ORO) stainings were performed as previously reported (23, 24). For ORO images, representative fields from each treatment were photographed using a 100× objective to count single intracellular LDs. Z-stacks of Nile Red–stained cells were performed using the Nikon Eclipse Ti inverted microscope equipped with Xcite 120 LED light source and Zyla sCMOS camera equipped with NIS-Element version 4 software. LD quantification was performed using FIJI according to a previously validated approach (23). LD distribution was calculated using the integral (area under the curve, or AUC). The integral for each LD group was calculated using trapezoidal numerical integration in GraphPad Prism.

### Western blotting

Protein lysates were prepared using RIPA buffer (J63306, Alfa Aesar) combined with 1× protease inhibitor cocktail (1862209, TFS) and 1× phosphatase inhibitor cocktail (P5726, Sigma Aldrich). Twenty micrograms of protein was separated and transferred using the Mini-PROTEAN TGX Stain-Free System (Bio-Rad Laboratories, Inc., SCR_008426). Membranes were blocked with 3% BSA and 1% Tween 20 in PBS for 30 minutes and then incubated overnight (with constant rocking) at 4°C with each primary antibody. Catalog numbers and dilutions of the antibodies used are listed in Supplementary Table S2. The next day, samples were incubated for 1 hour at room temperature with appropriate horseradish peroxidase–conjugated secondary antibodies (Cell Signaling Technologies Inc. at 1:5,000 dilution), followed by exposure to Clarity Western ECL Substrate Kit (Bio-Rad Laboratories, Inc.). Images were captured using the ChemiDoc Touch system (Bio-Rad) with stain-free technology for gel, membrane, and blot images, enabling total protein normalization.

### RNA isolation, RT-qPCR, and RNA sequencing analyses

Total RNA was extracted from cells overexpressing DGAT1 or from CAF isolated from AA or EA patients using the RNeasy Mini Kit (74106, Qiagen). For cDNA synthesis, 1 μg of total RNA was reverse transcribed using the iScriptTM cDNA Synthesis Kit (Bio-Rad), and 1 μL of cDNA template was added to IQ RealTime SYBR Green PCR Supermix (Bio-Rad) for RT-PCR. Relative quantification was calculated using the ΔΔCt method and was normalized to GAPDH. The DGAT1 primers DGAT1-forward (5′-CTT​GGT​GGT​ATC​CTC​CCT​CTA-3′) and DGAT1-reverse (5′-GCA​GGC​TTT​GCT​GCT​TTA​TC-3′) were purchased from Integrated DNA Technologies. For RNA sequencing (RNA-seq) studies, total RNA was cleaned using the RNeasy Plus Mini Kit (Qiagen 74134) and shipped to Novogene for poly A selection and library preparation using the NEBNext Ultra II RNA Library Prep Kit for Illumina (New England BioLabs E7770). Sequencing was performed with 2 × 150 bp reads on a NovaSeq 6000 PE150, followed by bioinformatics analysis. Read mapping was conducted using STAR v2.6.1d (RRID: SCR_004463), differential gene expression analysis was performed with the DESeq2 R package (1.42.0; RRID: SCR_000154), read counts were adjusted using the edgeR R package (4.0.16; RRID: SCR_012802), and significantly differentially expressed genes between groups with sufficient power were identified based on an adjusted *P* value ≤ 0.05. Gene Ontology (GO) was used for enrichment analysis and visualization of altered pathways. No codes were generated for the analysis of transcriptome data.

### Animal studies

Animal studies were approved by the Institutional Animal Care and Use Committee (IACUC) of NorthShore University Health System (# EH18-353). All mice in this study were maintained under constant environmental conditions in the Animal Research Facility with free access to food and water. A total of 100,000 epithelial cells (BPH1, LNCaP, or PC-3) were combined with 250,000 stromal cells (BHPrS1^EV^ or BHPrS1^DGAT1^) in neutralized rat tail collagen to make tissue recombinants and incubated at 37°C overnight. The recombinants were grafted under the kidney capsules of intact male CB17Icr/Hsd-SCID mice (Envigo) and supplemented with 25 mg testosterone via subcutaneously implanted testosterone pellets (21). One or two grafts were placed under the renal capsule of each kidney. Subcapsular BPH1 grafts grew *in vivo* for 8 weeks, LNCaP grafts for 6 weeks, and PC-3 grafts for approximately 4 weeks before euthanasia. The kidneys were harvested, measured, photographed, and fixed in formalin. Imaged kidneys were used to measure tumor growth on the kidney using the Fiji plugin in ImageJ (RRID: SCR_003070; ref. 25). Briefly, the grafts were imaged, and tumor length, width, and height were quantified using ImageJ (RRID: SCR_003070). Tumor volume was calculated using an ellipsoid formula as previously described (9, 21).

### Xenograft processing and immunohistochemical staining

After harvesting, the kidneys were cut in half, processed, and embedded in paraffin. Tissue sections were sliced at 4 μm for hematoxylin and eosin staining and immunohistochemistry (IHC). After deparaffinization and hydration, we performed antigen retrieval using an antigen unmasking solution (Vector Laboratories). For antibody visualization, the Vectastain Elite Kit (Vector Laboratories) was used according to the manufacturer’s instructions. The primary antibodies were incubated in a humidified chamber at 4°C overnight.

### Cytokine array

The expression of 105 secreted human cytokines in each of the engineered BHPrS1 cells was evaluated using an XL human cytokine antibody array kit (ARY022B, R&D Systems). Cytokine arrays were incubated overnight at 4°C with 500 μL of conditioned media, and the procedure was carried out according to the manufacturer’s instructions. After incubation with a detection antibody cocktail, antibody conjugation, and recommended washes, the immunoblots on the membrane were developed using the Chemiluminescent Substrate Reagent Kit (Bio-Rad). Signals on each array were detected using ChemiDoc Imaging software (Bio-Rad), and signal intensity was quantified using the Fiji plugin in ImageJ software (RRID: SCR_002285, version 1.53o; refs. 9, 25). Mean signal intensities were corrected by subtracting the median background intensities. Significant changes in cytokine secretion, upregulation (≥1.5-fold) or downregulation (≤0.5-fold), were determined by comparison with the controls for proteins with signal density values greater than 200 pixels (*P* < 0.05).

### Statistical analysis

Data are presented as the mean ± standard error of the mean (SEM), representing at least three independent experiments performed in triplicate. ANOVA and Tukey multiple comparisons *post hoc* tests were used to determine differences between multiple groups. The Student *t* test was used when comparing two groups, considering statistically significant differences at *P* < 0.05. This analysis was performed using GraphPad Prism (RRID: SCR_002798), version 10.

### Consent for publication

No personal data are presented.

## Results

### Racial differences in stromal DGAT1 expression show increased lipid storage response by CAFs from AA compared with EA patients with prostate cancer

**Figure 1.**
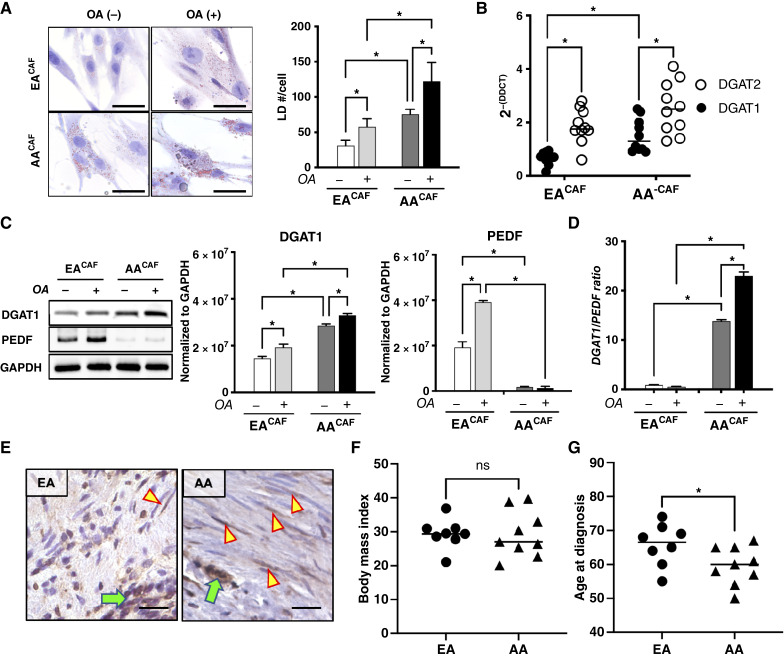
Racial DGAT1 expression differences in CAFs. **A,** Identification of LDs by ORO staining in cultured primary fibroblasts isolated from EA and AA cancer tissues (CAFs), *n* = 10/group (left). LD density (number of LDs/cell) was quantified and compared under OA exposure (OA±) and between racial cohorts (right). The black scale bars indicate that pictures were taken at the same magnification. **B,** Quantitative RT-PCR of DGAT1 expression was analyzed in CAFs from EA (*n* = 9) and AA (*n* = 10) patients with prostate cancer. **C,** DGAT1 Western blot of CAF (*n* = 10/group) cultured in the presence/absence of OA (left). Protein expression (densitometry normalized to GAPDH housekeeping gene) analysis showing DGAT1 (middle) and PEDF (right) responses to OA in EA and AA. **D,** The DGAT1/PEDF protein ratio was quantified in EA^CAF^ and AA^CAF^ cultured in the presence/absence of OA. **E,** IHC staining for DGAT1 in human prostate cancer tissues. Representative images showing a higher density of positive DGAT1-expressing stromal cells (yellow arrowheads) in AA vs. EA near cancer cells (green arrows). Scale bars, 10 μm. **F** and **G,** Body mass index and age data at the time of RALP of EA (*n* = 8) and AA (*n* = 9) patients with prostate cancer. For two groups, two-tailed, unpaired Student *t* test was used, and ANOVA was used for comparison between multiple groups, with significant comparisons identified by a Tukey test. *, *P* < 0.05.

CAFs accumulate more neutral lipids in the form of LDs than normal fibroblasts (19). Obesity and metabolic syndrome are associated with a higher risk of prostate cancer, particularly in AA men (20, 26). We and others have previously shown that excess lipid storage in cancer tissues is redistributed to other mesenchymal cells, such as CAFs in the TME (19, 27). To determine whether racial differences in lipid storage are seen in prostate CAFs, ORO staining was performed in primary CAFs isolated from patients with prostate cancer to assess LD status. Elevated basal LD density was observed in AA^CAF^ versus EA^CAF^ (Fig. 1A). Treatment with 100 μmol/L OA, a potent FA stimulator of LD formation, significantly increased LD accumulation in AA^CAF^ (Fig. 1A). DGAT1 and DGAT2 are rate-limiting enzymes that respond to excess FA, regulate triglyceride (TG) synthesis, and contribute to LD formation (DGAT1) and expansion (DGAT2) to prevent lipotoxicity. To explore the role of stromal DGAT1 and DGAT2 in prostate cancer racial disparities, we performed quantitative RT-PCR analysis of DGAT1 transcripts in AA^CAF^ and EA^CAF^. Both DGAT1 mRNA and protein levels were significantly higher in AA^CAF^ than in EA^CAF^ (>2 fold, *P* < 0.05; Fig. 1B and C). Although DGAT2 expression was higher than DGAT1, no significant racial differences were observed (Fig. 1B). Interestingly, DGAT2 levels were consistently higher than those of DGAT1 in both cohorts. Under FA overload with OA, DGAT1 protein expression increased in both racial groups, with a greater response in AA^CAF^ than in EA^CAF^ (Fig. 1C). Further examination of lipogenesis and lipolysis genes showed notably lower endogenous expression of the lipolysis factor PEDF in AACAF, resulting in a higher DGAT1/PEDF ratio that can shift the balance toward LD accumulation (Fig. 1D). IHC analysis of a small cohort of prostate cancer tissues revealed heterogeneous DGAT1 expression in both cancer cells (green arrows) and in the TME. A higher number of DGAT1+ stromal cells (yellow triangles) was observed in prostate cancer samples from AA patients than in those from EA patients (Fig. 1E). To investigate whether increased stromal DGAT1 in AA could be due to excess lipids such as obesity, we assessed several clinicopathologic characteristics in our patient cohorts from which CAFs were isolated (Supplementary Table S1). No difference in body mass index was found between EA and AA men (Fig. 1F), but AA patients were significantly younger than EA patients at the time of RALP (Fig. 1G). This aligns with previous reports that prostate cancer often occurs at a younger age in AA men compared with EA men. Overall, these lipogenesis-promoting changes in the TME involving DGAT1 may explain the racial differences in LD storage response in CAFs to FA overload (Fig. 1A).

### DGAT1 overexpression in prostate fibroblasts induces CAF activation

**Figure 2.**
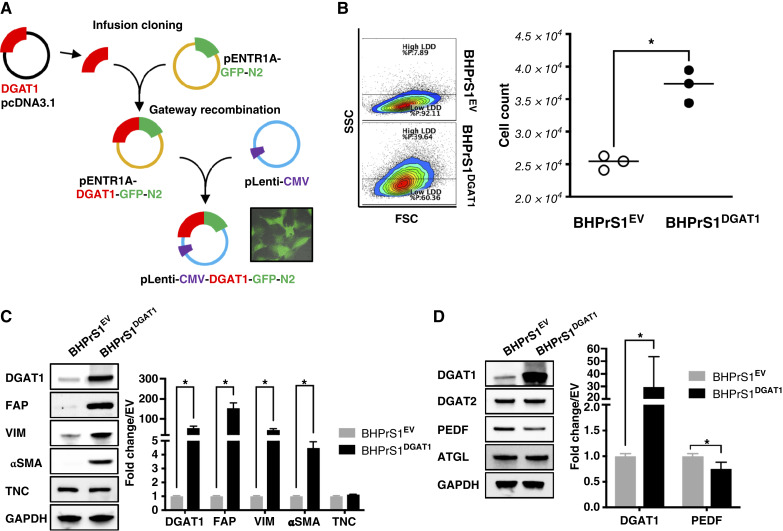
Increased DGAT1 expression induces prostate fibroblast activation. **A,** pLenti-CMV-DGAT1-GFP N2 cloning strategy (refer to “Materials and Methods”). Cells expressing green fluorescent protein (inner set). **B,** Analysis of the total number of cells (right) quantified by flow cytometry (left) and compared between BHPrS1-expressing DGAT1- BHPrS1^DGAT1^ and control cells- BHPrS1^EV^. *n* = 6, *, *P* < 0.05. **C,** Western blot analysis showing expression of CAF markers in BHPrS1^EV^ and BHPrS1^DGAT1^ cells. **D,** Western blot analysis of LD-regulating genes in BHPrS1^DGAT1^ and BHPrS1^EV^ cells. Densitometric analysis of Western blots is indicated and represents experiments performed in triplicate. Two-group comparisons were analyzed by two-tailed, unpaired Student *t* test; multiple-group comparisons were analyzed by one-way ANOVA with Tukey *post hoc* test. *, *P* < 0.05.

The mechanisms that regulate CAF activation in a lipid-rich environment are unknown. To better understand its role in CAF biology, we generated a fused DGAT1/GFP lentiviral vector and transduced it into benign human prostate fibroblasts (BHPrS1), creating BHPrS1^DGAT1^ and a control cell line, BHPrS1^EV^ (Fig. 2A). The proliferative capacity of serum-starved, synchronized BHPrS1^DGAT1^ cells compared with BHPrS1^EV^ cells was measured by flow cytometry. A significant (*P* < 0.01) increase in cell count was observed in BHPrS1^DGAT1^ cells compared with BHPrS1^EV^ cells (Fig. 2B).

Compared with control cells, significantly higher DGAT1 (∼50-fold) expression was observed in the engineered BHPrS1^DGAT1^ cells (Fig. 2C). To determine whether BHPrS1 cells with increased DGAT1 expression acquire a CAF-like phenotype, we examined the protein levels of putative CAF markers (Fig. 2C). Several CAF markers showed significantly increased levels of fibroblast activation protein (FAP; 150-fold), α-smooth muscle actin (αSMA; 50-fold), and vimentin (50-fold, *P* < 0.01) in DGAT1-expressing BHPrS1 cells. Minimal changes were observed in tenascin-c (TN-C), platelet-derived growth factor receptor α (PDGFRα), and fibroblast stimulating protein-1 (FSP-1). These findings suggested that elevated DGAT1 levels in stromal cells may regulate CAF marker expression and contribute to CAF activation.

In addition to DGAT1 effects on fibroblast activation, we characterized the protein levels of key molecules responsible for the balance of the lipogenesis/lipolysis ratio in BHPrS1^DGAT1^ to determine whether changes induced by DGAT1 could affect the expression of LD-regulating factors (Fig. 2D). Overexpression of DGAT1 in BHPrS1 did not affect DGAT2 expression, another DGAT isoform enzyme involved in LD biogenesis. However, the expression of lipolysis ATGL activating factor PEDF significantly decreased (*P* < 0.05) in BHPrS1^DGAT1^ cells without affecting the levels of ATGL (Fig. 2D). These data suggest that altering lipogenesis factors such as DGAT1 could regulate lipogenesis while suppressing lipolysis, thus altering net lipid flux to potentially induce LD accumulation in fibroblasts.

### LD storage in BHPrS1^DGAT1^ decreases in response to a DGAT1 enzymatic inhibitor

**Figure 3.**
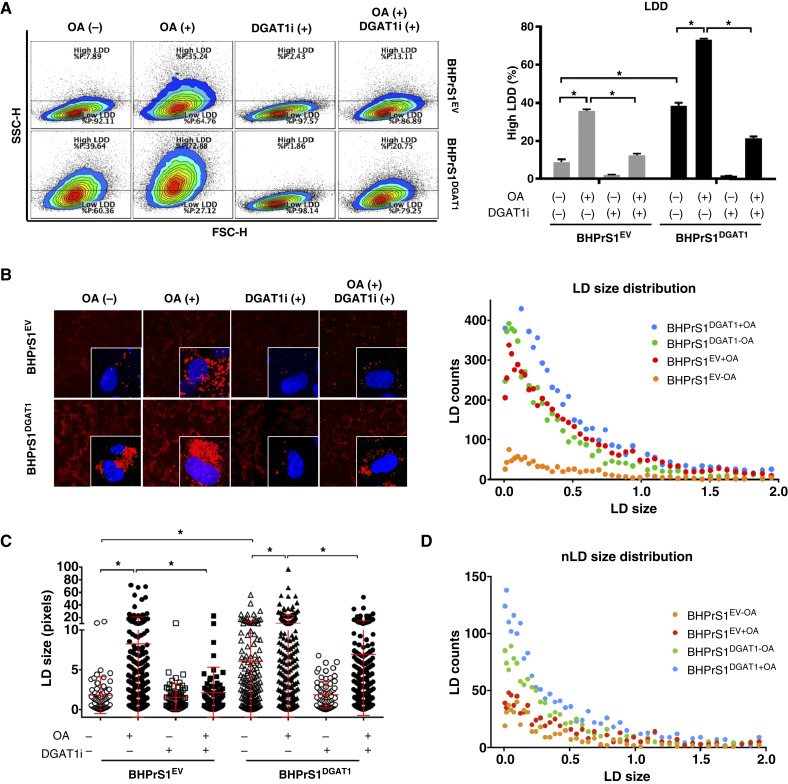
DGAT1 increases LD density in BHPrS1 cells. **A,** BHPrS1^DGAT1^ and BHPrS1^EV^ cells were exposed to OA and/or DGAT1 inhibitor (DGAT1i) *n* = 3 and harvested for flow cytometry. A dot plot of SSC (*y*-axis) vs. forward scatter (FSC; *x*-axis) of BHPrS1 cells shows the granularity distribution divided into two gates that included the LDD-low region, representing many cells under basal conditions (blue edges of the contour plot), and LDD-high regions. Analysis of LD density based on the percentage of cells in the LDD-high gate (right). Note: OA(−) plots are the same as those displayed in Fig. 2B for cell counts. **B,** Nile Red (red) staining to visualize LD content was performed in BHPrS1^DGAT1^ and BHPrS1^EV^ cells (*n* = 3) exposed to OA and/or DGAT1i and imaged by confocal microscopy (left). Scatter dot plot (right) of LD size and count distribution obtained from ALDQ analyzed images BHPrS1^DGAT1^ (green: −OA, blue: +OA) and BHPrS1^EV^ (orange: −OA, red: +OA). **C,** Dot plot of LD size from BHPrS1^DGAT1^ and BHPrS1^EV^ exposed to a combination of OA and DGAT1i. **D,** Scatter dot plot of nLD size and count distribution from BHPrS1^DGAT1^ and BHPrS1^EV^ cells exposed to OA. Two-group comparisons were analyzed by two-tailed, unpaired Student *t* test; multiple-group comparisons were analyzed by one-way ANOVA with Tukey *post hoc* test. *, *P* < 0.05.

Given that DGAT1 overexpression increases the lipogenesis/lipolysis ratio (Fig. 2D) in BHPrS1 cells, we measured the lipid content in BHPrS1^EV^ and BHPrS1^DGAT1^ cells using a simple method for the detection and quantification of neutral lipid accumulation by flow cytometry (10). Baseline levels were established in BHPrS1^EV^ cells in the absence of any external stimuli (Fig. 3A). Using this baseline, two arbitrary gates: high LD density (LDD) and low LDD were selected based on cellular granularity [SSC-H axis; Fig. 3A (left)]. In both cell lines, a significant increase in high LDD was observed in the presence of OA after 48 hours (*P* < 0.05) compared with their respective baseline levels (Fig. 3A). The addition of a selective DGAT1 (A922500) enzymatic activity inhibitor (DGAT1i) decreased the high LDD (*P* < 0.05) and OA-induced effects (*P* < 0.05) in both cell lines. To visualize and quantify LD size distribution under the same conditions, Nile Red staining was performed, revealing a significant increase in the accumulation of smaller LD in BHPrS1^EV^ cells with OA (red dots, Fig. 3B and red bar in Supplementary Fig. S1A) compared with the basal condition (orange dots/bar). LD changes (size and frequency) induced by DGAT1 in BHPrS1 cells (green dots) mirrored OA-induced effects on BHPrS1^EV^ cells (red dots). BHPrS1^DGAT1^ cells (blue dots) were still responsive to OA exposure, resulting in higher LD counts (Fig. 3B and blue bar in Supplementary Fig. S1A). Elevated DGAT1 expression led to the formation of larger LDs under basal conditions in BHPrS1^DGAT1^ than in BHPrS1^EV^. The comparison of LD size between BHPrS1^EV^ and BHPrS1^DGAT1^ under OA exposure (blue vs. red dots) was not statistically significant. However, the LD size response to OA (compared with OA−) was more pronounced in BHPrS1^EV^ (orange vs. red dots) than the change in LD size (OA− vs. OA+) observed in BHPrS1^DGAT1^ cells (green vs. blue dots). Blocking DGAT1 enzymatic activity with the small-molecule inhibitor A-922500 in BHPrS1^DGAT1^ cells reduced LD size to basal levels seen in BHPrS1^EV^ (Fig. 3C). Nuclear LD accumulation displayed a size distribution similar to that of the cytoplasmic LDs (Fig. 3D; Supplementary Fig. S1B). These findings demonstrated that DGAT1 overexpression alone can increase LD storage in prostate fibroblasts under basal conditions. Enzymatic inhibition of DGAT1 restored LD storage to basal levels in stromal cells with high DGAT1 expression.

### BHPrS1^DGAT1^ cells induce prostate cancer tumor growth and invasion *in vivo*

**Figure 4.**
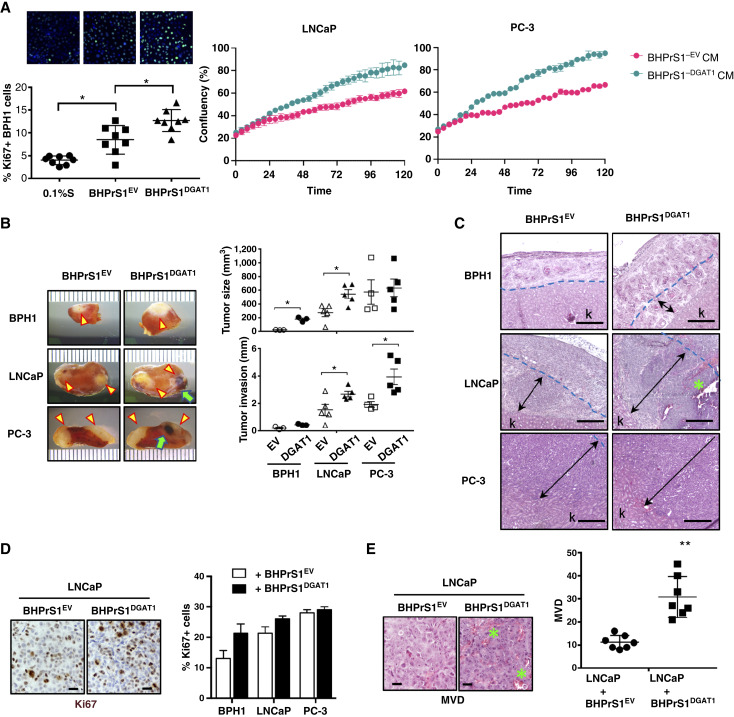
BHPrS1^DGAT1^ cells promote prostate cancer (PCa) cell tumorigenicity *in vivo*. **A,** Left, BPH1 cells were exposed to either 0.1% serum or conditioned media collected from BHPrS1^EV^ or BHPrS1^DGAT1^, fixed, and stained (top) for the proliferation marker Ki67 (green) and nuclear DAPI (blue). Quantification of Ki67-positive cells (bottom). *, *P* < 0.05. (right) Proliferation of LNCaP and PC-3 cells exposed to BHPrS1^EV^ or BHPrS1^DGAT1^ CM for 5 days using the Incucyte live-cell imaging system. **B,** PCa cells BPH1, LNCaP, and PC-3 were recombined with BHPrS1 cells expressing DGAT1 and empty vector (EV) control cells and grafted under the kidney capsule of SCID mice. Tumor growth (yellow arrowheads) and hemorrhagic features (green arrows) are depicted. Tumor growth (size in mm^3^) and invasion (distance from the kidney surface to the invasion front in mm) were quantified. *n* ≥ 3, *, *P* < 0.05. **C,** Hematoxylin and eosin (H&E) show areas of hemorrhage (indicated by green asterisks), the blue dotted line represents the renal surface (before tumor formation), and the black arrows indicate the distance of invasion from the capsule to inside the renal parenchyma. **D,** IHC (Ki67) evaluation of kidney xenografts (top) and analysis of the percentage of Ki67+ cells (bottom). **E,** H&E staining showing the microvasculature in LNCaP kidney xenografts (top) and quantification (bottom) of MVD. Scale bars, 10 μm. Two-group comparisons were analyzed by two-tailed, unpaired Student *t* test; multiple-group comparisons were analyzed by one-way ANOVA with Tukey *post hoc* test. **, *P* < 0.01.

DGAT1 overexpression induced CAF activation and lipid storage in BHPrS1 cells (Figs. 2C and 3), and biological features associated with potential functional facilitators of tumor progression. To assess whether higher stromal DGAT1 levels can influence epithelial cell proliferation through paracrine signaling, we collected conditioned medium (CM) from BHPrS1^DGAT1^ and BHPrS1^EV^. BPH1 (a genetically initiated prostate epithelial cell line with locally aggressive potential), LNCaP, and PC-3 (metastatic) cells were exposed to CM collected from BHPrS1^DGAT1^ and BHPrS1^EV^ or basal control conditions [0.1% bovine serum albumin (BSA)] for 24 hours. Proliferation, measured by Ki67 staining, showed that BPH1 cells exposed to BHPrS1^EV^ CM had more Ki67+ cells (*P* < 0.05) than control cells (Fig. 4A). The percentage of Ki67+ cells increased significantly (*P* < 0.05) when exposed to BHPrS1^DGAT1^ CM compared with BHPrS1^EV^ CM (Fig. 4A). Similar *in vitro* observations of cell proliferation over time were observed in the aggressive prostate cancer cell lines LNCaP and PC-3 (Fig. 4B). Treatment with DGAT1i reduced BHPrS1^DGAT1^-induced proliferation in these cells (Supplementary Fig. S1C). These findings suggest that factors secreted by BHPrS1^DGAT1^ cells can significantly promote the proliferation of prostate epithelial cells.

To extend our *in vitro* observations and understand the significance of stromal DGAT1 in prostate cancer tumor growth and/or invasion *in vivo*, we conducted subrenal capsule xenografts in SCID mice. In this study, recombinants of prostate epithelial cells—BPH1 (premalignant), LNCaP (low-tumorigenic with metastatic potential), or PC-3 cells (highly metastatic) with stromal cells: BHPrS1^EV^ or BHPrS1^DGAT1^ BHPrS1^DGAT1^ were grafted under the kidney capsule of adult SCID mice (Fig. 4B). The presence of BHPrS1^DGAT1^ significantly enhanced tumorigenicity in BPH1 and LNCaP cells compared with BHPrS1^EV^, leading to notable increases in tumor growth and invasion (*P* < 0.05). Overall, the size of PC-3 tumors was unaffected by BHPrS1^DGAT1^, but these cancer cells exhibited greater invasive capacity than BHPrS1^EV^ cells exposure (Fig. 4B). Histologically, these cells retained their lineage-specific malignant features, with BPH1 cells transforming into adenocarcinoma with multifocal squamous differentiation, and increased hemorrhagic areas observed in LNCaP and PC-3 cells (Fig. 4C). Compared with controls with BHPrS1^EV^, both LNCaP and PC-3 cells combined with BHPrS1^DGAT1^ showed increased invasion into the renal parenchyma (Fig. 4B and C). IHC analysis confirmed that GFP-positive BHPrS1 cells persisted in the tumor stroma for several weeks after implantation (Supplementary Fig. S1D). Tumors also demonstrated enhanced proliferation of BPH1 and LNCaP cells, as assessed by Ki67 staining in the presence of BHPrS1^DGAT1^ cells, correlating with the observed increase in tumor size (Fig. 4D). Additionally, higher microvessel density (MVD; Fig. 4E) and CD31 staining index (Supplementary Fig. S1E) were observed in LNCaP tumors with BHPrS1^DGAT1^ (*P* < 0.05) compared with those with BHPrS1^EV^. These findings suggest that increased stromal DGAT1 expression promotes prostate cancer tumorigenicity by enhancing cancer cell proliferation, invasion, and angiogenesis.

### BHPrS1^DGAT1^ cells secrete protumorigenic factors via ERK pathway activation

**Figure 5.**
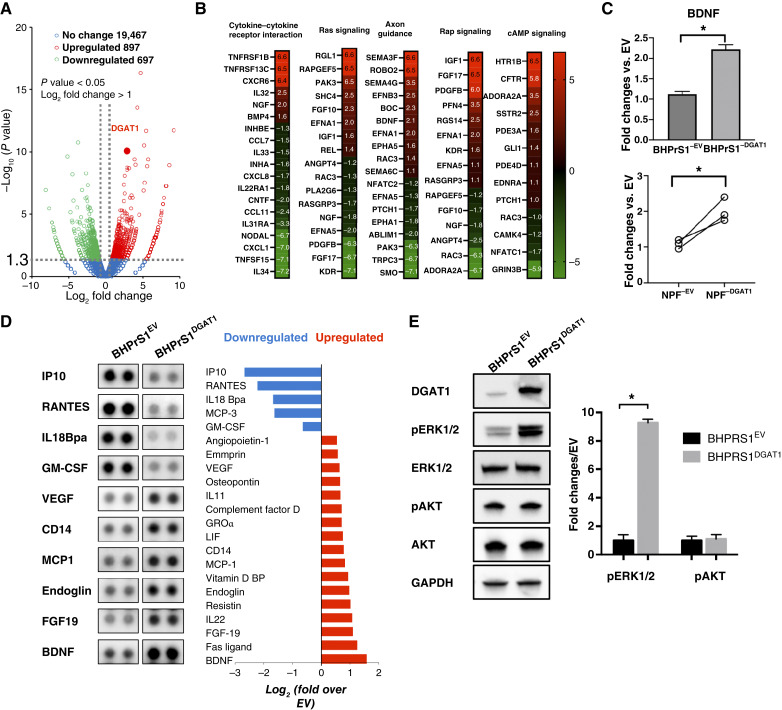
Molecular differences of engineered cell lines. **A,** Volcano plot of RNA-seq data from BHPrS1^DGAT1^ cells compared with BHPrS1^EV^ control cells. Fold change (Log_2_ = >1) calculated comparing BHPrS1^DGAT1^ to BHPrS1^EV^. (red = upregulation, green = downregulation, blue = no change) DESeq2 R package (1.42.0; RRID: SCR_000154), *P* < 0.05 adjusted with the Benjamini and Hochberg method. **B,** Pathway analysis heatmap showing significant gene alterations (red = upregulation, green = downregulation) in BHPrS1^DGAT1^ cells compared with BHPrS1^EV^ control cells. clusterProfiler R package, *P* < 0.05. **C,** Top, qRT-PCR BDNF expression in BHPrS1^DGAT1^ vs. BHPrS1^EV^. Bottom, primary benign prostate fibroblasts isolated from prostate tissues were transiently transfected with the DGAT1 or empty vector (EV) plasmids. Expression of BDNF was assessed by qRT-PCR and compared between groups *, *t* test, *P* < 0.05. **D,** Conditioned media were collected from BHPrS1^DGAT1^ and BHPrS1^EV^ cells for the assessment of secreted factors using a cytokine array. After normalization and background subtraction, data are presented as log_2_ fold change over BHPrS1^EV^. Blot of selected secreted factors (duplicates) with evident changes between BHPrS1^DGAT1^ and BHPrS1^EV^ groups are shown (left). Analysis of cytokines with significant changes (*t* test, *P* < 0.05) shows upregulated (red bars) and downregulated (blue bars) targets (right). **E,** Western blot analysis of downstream target pathways regulated in BHPrS1^DGAT1^ and compared with BHPrS1^EV^ cells. Densitometric analysis of Western blot images. *N* = 3, *, Two-group comparisons were analyzed by two-tailed, unpaired Student *t* test. *, *P* < 0.05.

Expression of CAF markers, as well as *in vitro* and *in vivo* effects (Figs. 2 and 3), suggest altered regulatory mechanisms induced by DGAT1 in BHPrS1^DGAT1^. To identify potential DGAT1 target genes in stromal cells, we isolated RNA from BHPrS1^EV^ and BHPrS1^DGAT1^ cells and performed RNA-seq analysis. Several differentially expressed genes were observed between BHPrS1^EV^ and BHPrS1^DGAT1^ groups. A volcano plot showing these differentially regulated genes in BHPrS1^DGAT1^ compared with BHPrS1^EV^ indicates the upregulation of 897 genes and downregulation of 697 genes (Fig. 5A). GO analysis revealed genes associated with several pathways, including cytokine regulation, axon guidance, and Ras, Rap, and cAMP signaling in BHPrS1^DGAT1^ cells compared with BHPrS1^EV^ (Fig. 5B). We have previously observed a unique secretome in AA^CAF^ that include higher BDNF secretion. The transcriptional expression of *BDNF* in BHPrS1^DGAT1^ observed in the RNA-seq data was validated by qRT-PCR [Fig. 5C (top)]. Additionally, *BDNF* expression was increased in primary normal prostate fibroblasts (*n* = 3) isolated from benign prostate tissues after transient transfection with the DGAT1 plasmid [Fig. 5C (bottom)]. These findings suggest that DGAT1’s protumorigenic effects could be mediated by paracrine signals from stromal cells.

To better understand the cross-talk between DGAT1-expressing BHPrS1 and cancer cells, we analyzed a panel of secreted factors (cytokines and chemokines). The CM from the engineered cell lines showed significant (*P* < 0.05) differences in secretion for each cell line (Fig. 5D). Some cytokines downregulated in BHPrS1^DGAT1^ include RANTES, IP10, IL18Bpa, and GM-CSF, whereas others were upregulated, such as VEGF, MCP1, FGF19, endoglin, CD14, and BDNF (Fig. 5D). Higher expression levels of some cytokines such as BDNF, MCP3, and IL22 was observed in the RNA-seq data, suggesting a potential novel transcriptional regulation by increased DGAT1 levels in activated fibroblasts (Fig. 5B). We previously reported higher BDNF secretion by AA^CAF^ compared with EA^CAF^. To better understand the downstream mechanisms of the BDNF-induced expression and secretion influenced by DGAT1, we examined the roles of phosphokinases phospho-ERK (pERK) and phospho-Akt (pAkt), which are known to be involved in BDNF regulation. Whereas pERK was significantly increased, no changes were observed in pAkt in BHPrS1^DGAT1^ cells compared with BHPrS1^EV^ (Fig. 5E). These findings demonstrate that fibroblasts with elevated DGAT1 levels possess a unique transcriptome and may have enhanced protumorigenic paracrine functions through modulation of the CAF secretome.

### DGAT1 inhibition differentially regulates secreted factors in AA^CAF^ versus EA^CAF^ and reduces CAF paracrine stimulatory effects

**Figure 6.**
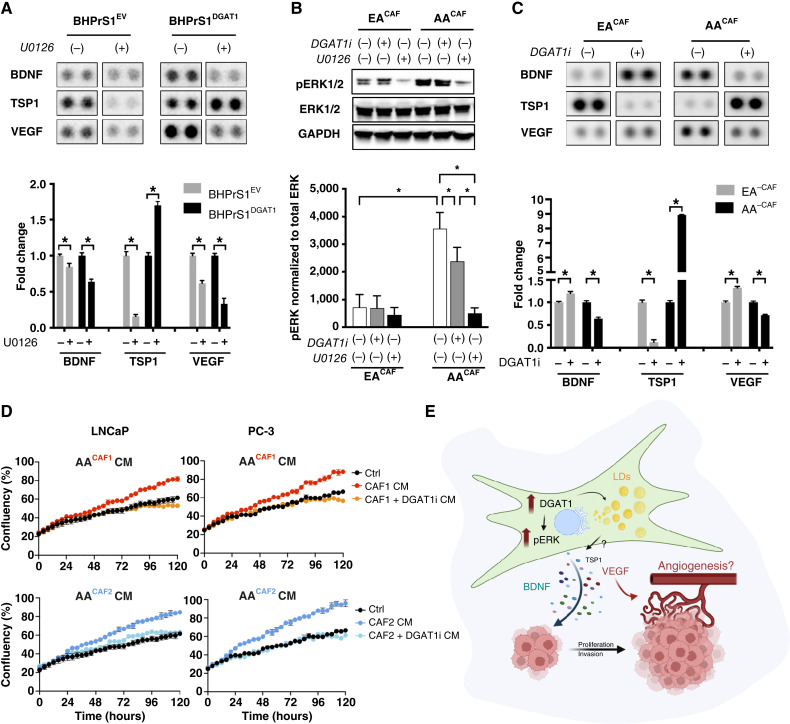
DGAT1 regulates BDNF in human prostate fibroblasts. **A,** Cytokine array of conditioned serum from BHPrS1^DGAT1^ and BHPrS1^EV^ ± U0126, technical replicates = 2, *P* < 0.05 (top). Quantification of cytokine array is shown as fold change over no U0126 treatment (bottom). **B,** Western blot (top) of pERK1/2 in high-DGAT1 expressing EA^CAF^ and AA^CAF^ cultured ± DGAT1i, *n* = 3. Changes in ERK activation was quantified and normalized to total ERK content (bottom), *P* < 0.05. **C,** Conditioned serum collected from high-DGAT1 expressing CAFs from AA and EA treated with ± DGAT1i for 72 hours was used for cytokine array. Densitometry values were normalized to background. Fold changes after DGAT1i exposure were calculated. Bars show selected secreted factors with significant differential racial response upon DGAT1 inhibition. Technical replicates = 2, *P* < 0.05. **D,** LNCaP and PC-3 cells were grown in the presence of two different AA^CAF^ CM treated with or without DGAT1i and compared with control cells cultured under basal conditions (see “Materials and Methods”). Cell confluency (each data point) was evaluated using Incucyte live-cell imaging over 5 days. **E,** Schematic illustration of CAF showing DGAT1 promotion of LD accumulation and regulation of secreted factors such as BDNF, VEGF, and TSP1 with potential roles in tumor growth, invasion, and angiogenesis. Multiple-group comparisons were analyzed by one-way ANOVA with Tukey *post hoc* test. *, *P* < 0.05. [**E,** Created in BioRender. Franco, O. (2026) https://BioRender.com/4jno6ge.]

To determine whether DGAT1 regulation of BDNF requires ERK activation, we collected conditioned media from BHPrS1^EV^ and BHPrS1^DGAT1^ cells treated with the ERK inhibitor 10 μmol/L U0126 for 72 hours. U0126 significantly reduced BDNF secretion (*P* < 0.05) in both cell lines (Fig. 6A). In addition to BDNF, several secreted factors that are regulated by the DGAT1–ERK axis were identified. Notably, proangiogenic/protumorigenic VEGF levels decreased upon ERK inhibition, whereas antiangiogenic TSP1 significantly increased in BHPrS1^DGAT1^ cells compared with BHPrS1^EV^ (Fig. 6A).

We demonstrated that DGAT1 regulates the secretome of BHPrS1^DGAT1^ cells via the ERK pathway. To determine whether similar regulation is present in CAFs and whether high DGAT1-expressing AA^CAF^ is associated with changes in ERK activation, we measured ERK phosphorylation in CAFs isolated from patients with prostate cancer self-identified as AA or EA. Western blot analysis showed slightly increased pERK1/2 in AA^CAF^ compared with EA^CAF^ (Fig. 6B). Next, because higher DGAT1 levels were observed in AA^CAF^ (Fig. 1C), we tested whether blocking DGAT1 affected pERK. Addition of DGAT1i lowered pERK levels in both CAF groups, with stronger effects in AA^CAF^ than in EA^CAF^. The total ERK levels remained unchanged between the groups (Fig. 6A). We then examined whether suppressing DGAT1 in AA^CAF^ influences the secretion of protumorigenic factors compared with EA^CAF^ (Fig. 6C). We found several protumorigenic secreted molecules that were downregulated by DGAT1 inhibition, showing a similar trend in both AA^CAF^ and EA^CAF^. Notably, upon DGAT1 inhibition, several cytokines responded differently among races (Fig. 6C). In untreated cells, higher levels of BDNF and VEGF and lower levels of TSP1 were present in AA^CAF^ than in EA^CAF^ (Fig. 6C). However, these cytokines exhibited an inverse response to DGAT1i, with BDNF and VEGF being downregulated in AA^CAF^ and upregulated in EA^CAF^. Notably, TSP1 levels increased in AA^CAF^ but decreased in EA^CAF^ after DGAT1i treatment. Similar results were observed when DGAT1 was knocked down in various CAF cell lines (Supplementary Fig. S2A and S2B). Next, to determine whether DGAT1 inhibition in CAF affects their proproliferative paracrine effects, we treated AA^CAF^ with DGAT1i, collected medium after 72 hours, exposed LNCaP and PC-3 cells, and monitored their proliferation over 5 days using live-cell imaging (Fig. 6D). Compared with cells treated with non-CM (black dots), those exposed to CM from AA^CAF^ showed increased proliferation (red and blue dots). Pretreating CAFs with DGAT1i reduced the growth-promoting effects of CM from AA^CAF^ (Supplementary Fig. S2C). These findings suggest that the lipogenic enzyme DGAT1 controls the secretion of protumorigenic factors by CAF in a race-specific manner and can block the protumorigenic paracrine effects driven by CAFs with high DGAT1 levels. Targeting stromal DGAT1 may have potential implications in personalized therapies.

## Discussion

The prevalence of obesity and hyperlipidemia is higher in AA men compared with EA men (28, 29), and abdominal obesity is often associated with aggressive prostate cancer (30, 31). Excessive lipid accumulation leads to ectopic storage in nonadipocytes and provides a reservoir of energy for cancer cells (32). Lipid metabolic reprogramming in CAFs, followed by excess lipid storage, may provide an essential niche for cancer to flourish. In the current study, the evaluation of DGAT1, an enzyme critical for TG storage in LDs, revealed a racial disparity in the mRNA and protein levels in CAFs isolated from prostate cancer tissues. We hypothesized that racial DGAT1 expression differences in CAFs may have significant consequences on CAF biology and contribute to the aggressive forms of prostate cancer in AA men.

Our analysis of primary fibroblasts obtained from prostate cancer tissues showed elevated basal DGAT1 levels in AA^CAF^ compared with EA^CAF^. Consequential upregulation of LD upon lipid overload exposure correlated with racial disparity in levels of DGAT1 expression in CAFs (Fig. 1). However, studies on lipid metabolism in prostate CAFs are limited. Adipose-derived stromal/stem cells can be converted into CAFs in obese patients with cancer and support tumorigenicity (33, 34). A high incidence of cancer is generally associated with an increased prevalence of obesity, especially in the AA population (35–37). The molecular mechanisms regulated by a lipid-rich TME in AA men that support prostate cancer progression remain unclear. By increasing DGAT1 levels in the benign prostate fibroblast BHPrS1, stromal LD biogenesis and storage were stimulated. In BHPrS1^DGAT1^, increased expression of DGAT1 resulted in a significant decrease in the ATGL-binding protein PEDF. CAFs expressed negligible amounts of PEDF (24). Racial differences in PEDF levels observed in CAFs may alter ATGL function in lipolysis and contribute to DGAT1-induced lipogenesis, favoring stromal LD accumulation. Lipid-laden fibroblasts, referred to as lipofibroblasts, were recently identified in the TME of human lung tumors using single-cell transcriptome studies (38–40). It is increasingly accepted that CAFs are composed of a heterogeneous population of cells with different and unique functions. However, functional studies are required to fully determine the characteristics and relevance of distinct clusters. Our IHC observations suggested cluster expression within the CAF in the TME. Whether DGAT1 induces reprogramming in a subset of prostate fibroblasts and induces the generation of a population of stromal cells similar to lipofibroblasts is unknown.

In addition to serving as a source of energy (FA exchange) and LD storage, DGAT1 supported CAF activation and BHPrS1^DGAT1^ cells display a unique transcriptome and secretome. BHPrS1^DGAT1^ showed increased FAP, a key marker of CAFs, with multifaceted roles in promoting invasion and metastasis. Several groups have explored strategies to target FAP, including for immunotherapy (41–43). The translational (diagnostic or therapeutic) utility of FAP for racial stratification of patients with prostate cancer has not been studied. We also observed an increasing trend in Tenascin C (TNC) expression, another CAF marker, in BHPrS1^DGAT1^ cells (44, 45). TNC levels are higher in AA^CAF^, and its expression is associated with increased MVD and tumor-associated macrophage infiltration, inducing significant stromal remodeling in the TME compared with EA^CAF^ (46, 47). These results indicated that a novel pathway for CAF activation is linked to lipid reprogramming induced by DGAT1.

Several studies have emphasized the importance of paracrine interactions between CAFs and cancer cells during cancer progression. TME composition and the profile of secreted factors have been shown to be highly variable in AA patients compared with EA patients (9). Our study demonstrated that DGAT1-mediated LD biogenesis in fibroblasts represents a central metabolic checkpoint that coordinates the production of protumorigenic secreted factors, which significantly enhanced *in vitro* proliferation (Ki67) of prostate cancer cells (Fig. 4). By esterifying excess FFAs into neutral lipids, DGAT1 limits lipotoxic stress while simultaneously promoting a transcriptional and secretory program enriched in cytokines, chemokines, and growth factors, including BDNF, VEGF, IL11, CCL2 (MCP-1), CCL5 (RANTES), CXCL1 (GROα), and LIF. These factors converge on complementary signaling pathways in cancer cells, most prominently STAT3, MAPK/ERK, and PI3K/AKT, to enhance proliferation, survival, epithelial–mesenchymal transition (EMT), angiogenesis, immune evasion, and therapy resistance. For example, IL11 and LIF activate JAK/STAT3 signaling to reinforce tumor cell survival and stemness (48, 49); CCL2 and CCL5 enhance tumor–immune cross-talk and metastatic dissemination; and CXCL1 promotes invasion and stress adaptation (50, 51). Together, these DGAT1-regulated factors establish a metabolically primed stromal niche that amplifies epithelial tumor aggressiveness through coordinated paracrine signaling, thereby linking lipid metabolic rewiring in CAF to tumor progression. In addition, BHPrS1^DGAT1^ cells promoted increased invasion, angiogenesis, and recruitment of inflammatory infiltrates *in vivo*, favoring tumorigenicity of prostate cancer cell lines. PEDF, a key inhibitor of angiogenesis in prostate stromal cells (52, 53), was found to be significantly downregulated in BHPrS1^DGAT1^ cells compared with BHPrS1^EV^ cells. Enhanced MVD and immune inflammatory infiltrates in AA patients with cancer is supported by racial disparities in the expression of proangiogenic factor VEGF and proinflammatory cytokine IL6 (12). Reciprocal regulation of VEGF and TSP-1 by DGAT1 in our engineered cell lines reflected an identical relationship in patient fibroblasts (CAFs), emphasizing the potential racial disparity in stromal biology. BDNF, a member of the neurotrophin family, has potent survival and differentiation functions and provides guidance for cells of the central and peripheral nervous system (54, 55). Racial differences in BDNF have been observed in other diseases such as obesity, dementia, and human immunodeficiency virus–related cognitive disorders (16, 17). Tumor epithelial cells are thought to be the main source of neurotrophins; however, our data highlight a probable role for a subpopulation of stromal cells. CAF-derived BDNF can mediate tumor progression in AA by activating the PI3K/Akt pathway in cancer cells and promoting proliferation and motility (9). BDNF can also regulate the vasculature and act as a proangiogenic factor by increasing TrkB+ endothelial progenitor cells and hypoxia-inducible factor-1α–mediated VEGF (56, 57). We identified a novel differential racial regulation of VEGF, TSP-1, and BDNF via the ERK1/2 signaling pathway in the prostate stroma. Abrogation of DGAT1 with a specific inhibitor exerted diverse effects on several secreted factors in CAFs associated with racial differences. DGAT1 inhibition had a beneficial effect by reducing BDNF secretion in AA^CAF^ while significantly increasing its expression in EA^CAF^. This potential racially associated deleterious effect suggests that unknown underlying differences must be identified and considered for personalized approaches for treating prostate cancer. Although socioeconomics, environment, and culture play major roles, there are inherited genetic variations that may also play a role in drug response and adverse drug reactions. Race-based pharmacogenetics is currently challenging, and the need for a better understanding of biological responses has been identified as the key to creating personalized drugs with greater efficacy and safety.

Overall, our study demonstrates that there is a racial disparity in stromal DGAT1 expression in patients with prostate cancer. CAFs isolated from patients with prostate cancer and our engineered cell line, BHPrS1^DGAT1^, showed that DGAT1 is involved in CAF phenotypic activation, and our *in vivo* data demonstrate that increased stromal DGAT1 promotes tumor growth and invasion. We identified a set of DGAT1-target secreted factors in BHPrS1^DGAT1^ cells that were regulated in an ERK1/2-dependent fashion, which contribute to tumor progression (Fig. 6E). The differential response to DGAT1 inhibition in CAF from AA versus EA warrants further investigation of CAF biology and emphasizes the need for personalized approaches based on racial stratification.

## Supplementary Material

Table S1 Clinico-pahtological data of the patient cohort.

Table S2 List of antibodies used in the study

Figure S1 LD quantitation (AUC), PCa cell proliferation and IHC stainings

Figure S2 DGAT1 silencing validation and effects on secretome and PCa cells proliferation.

## Data Availability

RNA-seq data discussed in this publication have been deposited in NCBI’s Gene Expression Omnibus and are accessible through GEO series accession number GSE319645. The raw data generated in this study are available upon request to the corresponding author. Data presented in the article will be available online and upon request.
