# Supplementary figures and images for: DGAT1 Drives Racially Divergent Fibroblast Activation via ERK1/2-Dependent Tumorigenic Signaling in Prostate Cancer
Source: Cancer Res Commun. 2026 Jun 4;6(6):1305–18. doi: 10.1158/2767-9764.CRC-25-0701 (PMC13234498; doi:10.1158/2767-9764.CRC-25-0701)

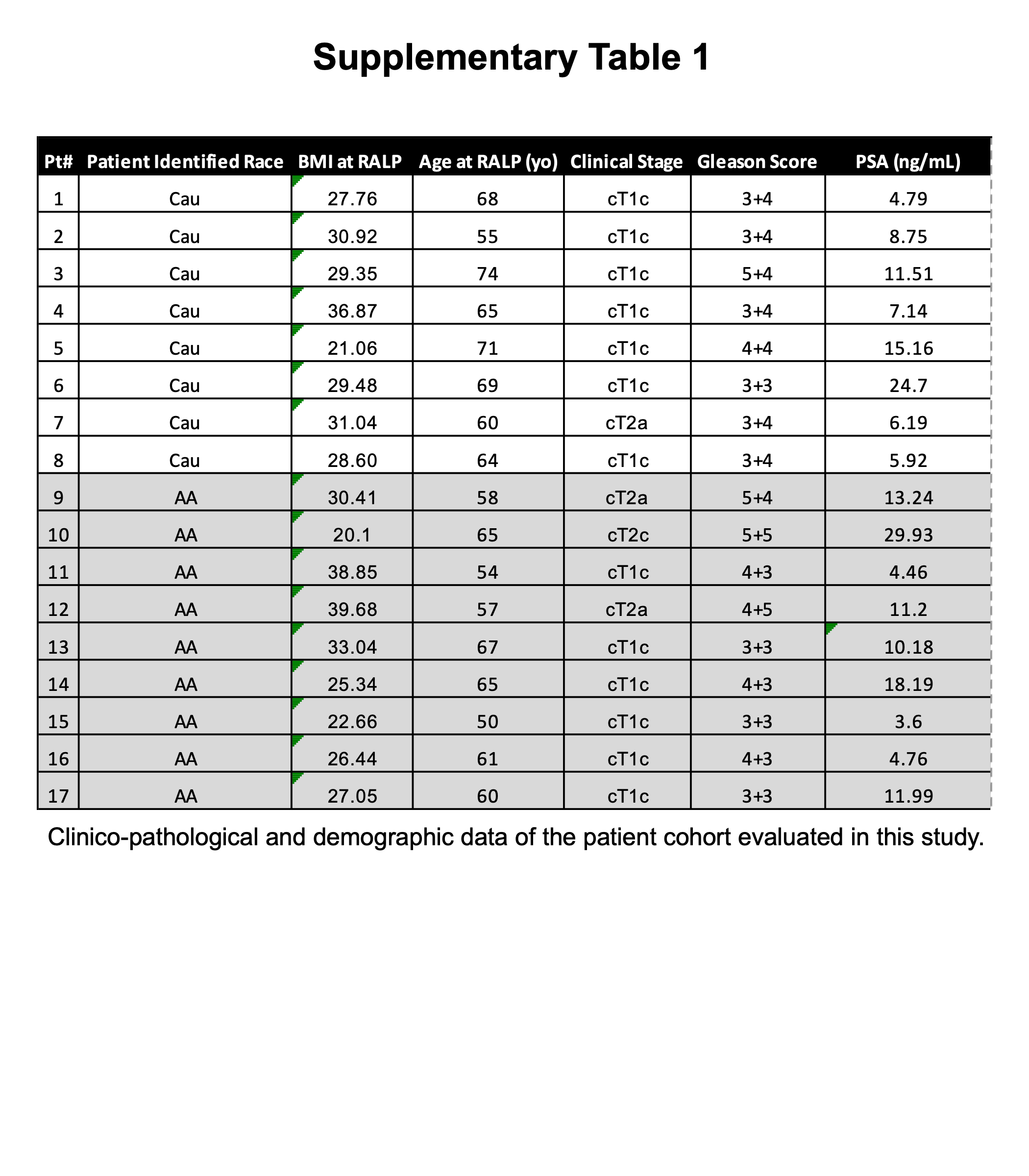

Supplement: Table S1 — Clinico-pahtological data of the patient cohort. [file crc-25-0701_table_s1_suppst1.png]

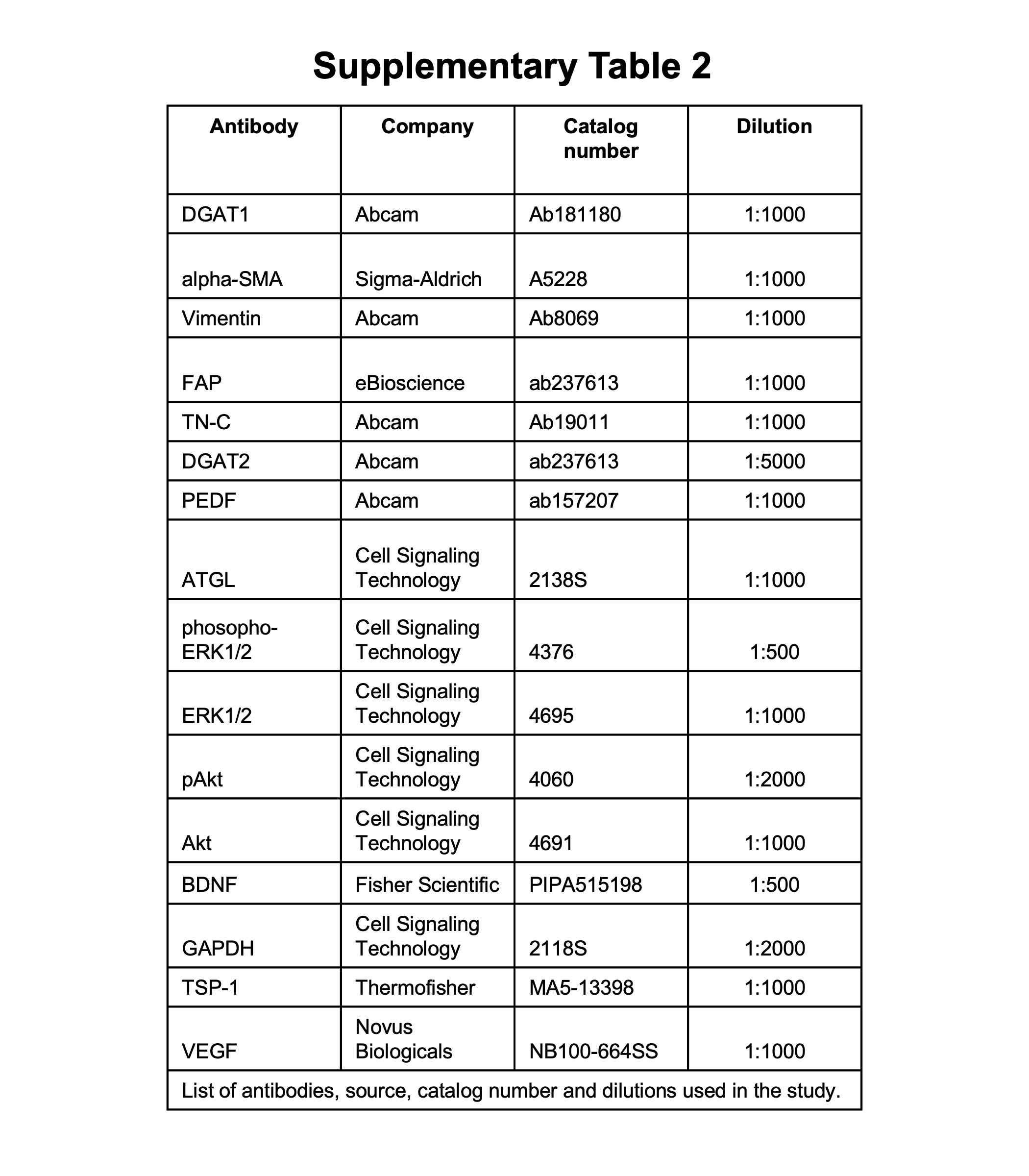

Supplement: Table S2 — List of antibodies used in the study [file crc-25-0701_table_s2_suppst2.png]

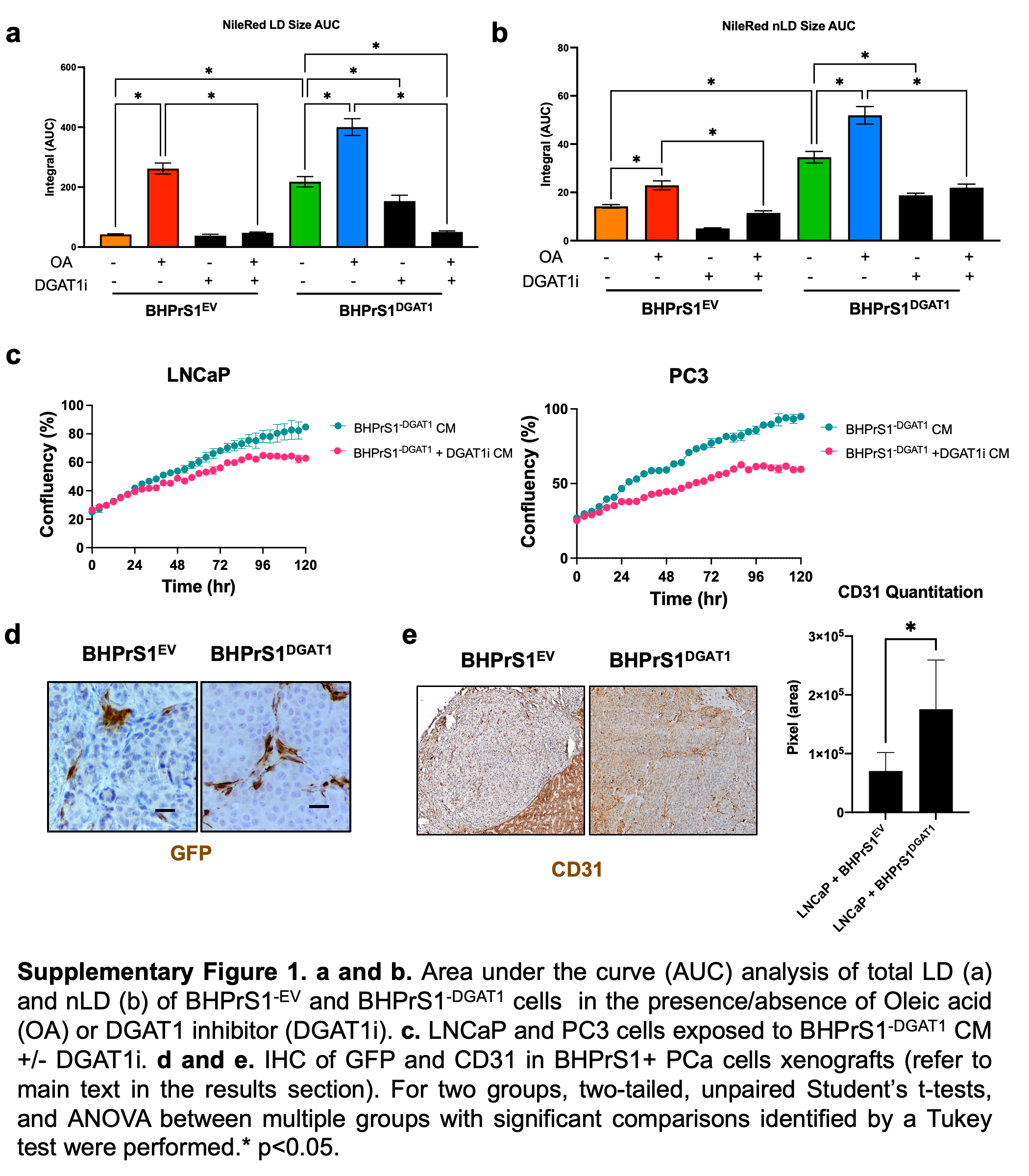

Supplement: Figure S1 — LD quantitation (AUC), PCa cell proliferation and IHC stainings [file crc-25-0701_figure_s1_suppsf1.png]

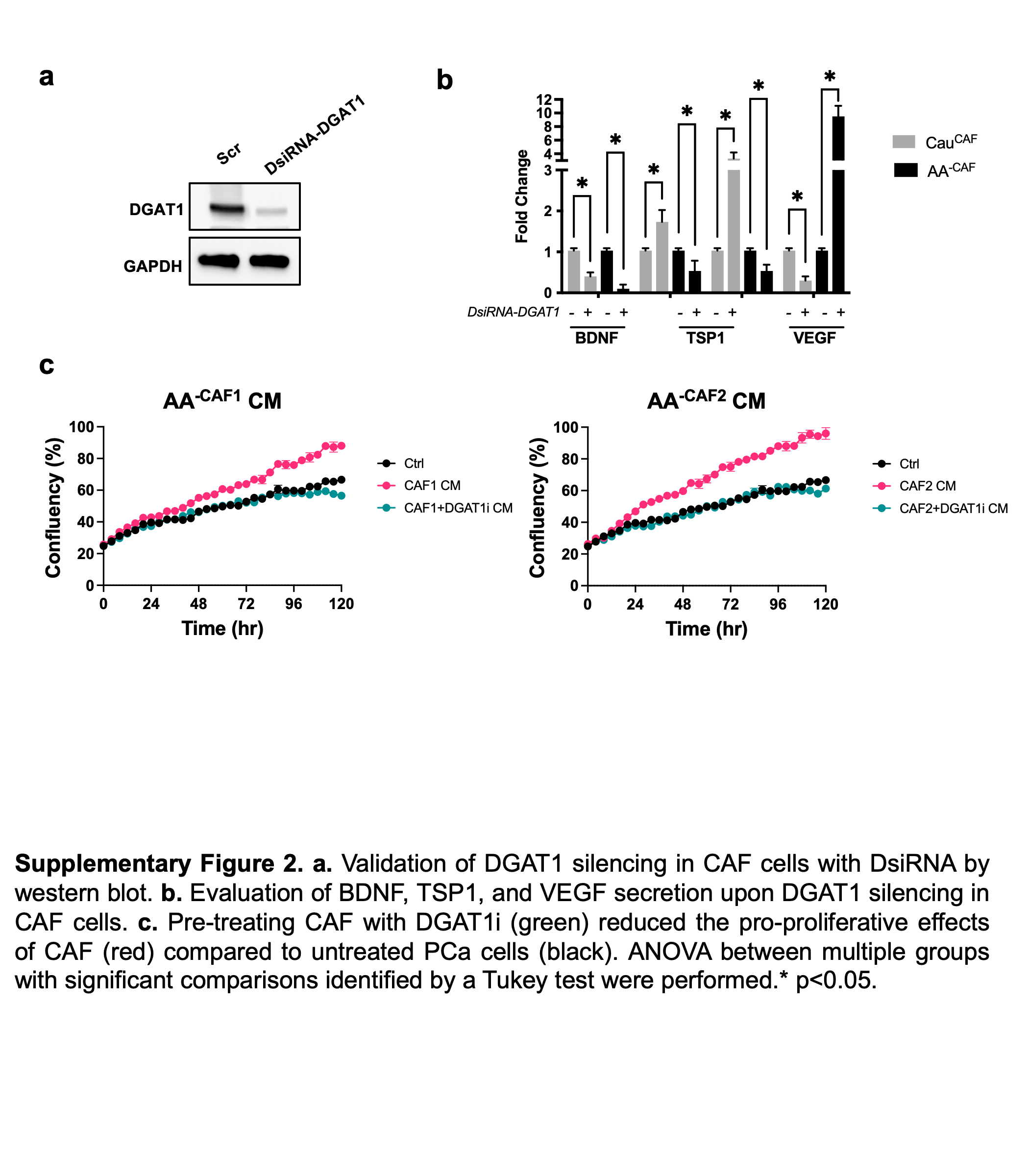

Supplement: Figure S2 — DGAT1 silencing validation and effects on secretome and PCa cells proliferation. [file crc-25-0701_figure_s2_suppsf2.png]
